# Identification of Notch pathway-related biomarkers in patients with idiopathic pulmonary fibrosis

**DOI:** 10.1371/journal.pone.0339287

**Published:** 2026-01-02

**Authors:** Shiyuan Yang, Yu Bao, Hailan Zhao, Chunlai Zhang, Yezhen Wang, Ke Li, Puguang Li, Wei Zhang, Xue Zhu

**Affiliations:** 1 First Clinical Medical School, Shandong University of Traditional Chinese Medicine, Jinan City, Shandong Province, China; 2 Affiliated Hospital of Tianjin University of Traditional Chinese Medicine, Tianjin University of Traditional Chinese Medicine, Tianjin City, China; 3 Laboratory medicine department, Shandong University of Traditional Chinese Medicine Affiliated Hospital, Jinan City, Shandong Province, China; 4 Department of Pharmacy, Shandong University of Traditional Chinese Medicine Affiliated Hospital, Jinan City, Shandong Province, China; Brigham and Women's Hospital, UNITED STATES OF AMERICA

## Abstract

The involvement of Notch pathway-related genes (NPRGs) in idiopathic pulmonary fibrosis (IPF) remains inadequately understood. This study identified novel NPRG-associated biomarkers in IPF through integrated analysis of the GSE28042 dataset and NPRG gene sets, with the goal of uncovering potential therapeutic targets. Initially, 7 overlapping candidate genes were identified by intersecting 1,361 differentially expressed genes (DEGs) between IPF and control samples, 4,883 key module genes associated with IPF, and 428 known NPRGs. Four biomarkers—*IL4*, *PLXND1*, *NBEA*, and *GATA2*—were prioritized using machine learning methods. Immune infiltration analysis, conducted with the CIBERSORT algorithm (v2.0.4), revealed that *IL4*, *NBEA*, and *GATA2* were significantly positively correlated with resting dendritic cells and negatively correlated with follicular helper T cells. Additionally, drug target prediction and pathway enrichment analyses suggested potential associations between these biomarkers and oxidative stress-related pathways. RT-qPCR validation using human blood samples confirmed significant down-regulation of *IL4* and *NBEA* while *PLXND1* was significantly up-regulated in patients with IPF compared to healthy controls. These biomarkers may contribute to the pro-fibrotic microenvironment, and their dysregulation is linked to the pathogenesis of pulmonary fibrosis. In summary, the identified NPRG-related biomarkers hold diagnostic potential for IPF. with further research needed to clarify their functional roles and assess their viability as therapeutic targets or as consequences of the fibrotic process.

## 1. Introduction

Idiopathic pulmonary fibrosis (IPF) is a distinct form of interstitial inflammation limited to the lungs and represents the most common type of idiopathic interstitial pneumonia (IIP) [1]. Clinically, IPF is characterized by progressively worsening dyspnea and a significant decline in lung compliance, which lead to severe structural damage and fatal outcomes, ultimately resulting in respiratory failure and death [2]. The onset of IPF is linked to genetic and environmental factors, comorbidities, and viral infections and is closely associated with aging, oxidative stress, and epithelial-to-mesenchymal transition (EMT) mechanisms [3,4]. The median survival time after an IPF diagnosis is only 3–5 years, with a poor prognosis and high mortality [5]. Diagnosing IPF presents substantial challenges, primarily due to subjective variability in interpreting lung imaging and the nonspecific nature of early symptoms (such as coughing and breathing difficulties), which are often mistaken for other interstitial lung diseases. This leads to delayed or incorrect diagnoses. As a refractory disease, IPF currently has limited treatment options. Although antifibrotic drugs, such as nintedanib and pirfenidone, can slow disease progression, there is no cure available, other than lung transplantation [6,7]. Therefore, identifying novel biomarkers and elucidating the molecular mechanisms underlying IPF will aid in improving diagnosis, guiding targeted drug development, and enhancing clinical management of the disease.

The Notch signaling pathway is a highly conserved cellular mechanism that governs embryonic development, maintains tissue homeostasis, and regulates cell proliferation through interactions between Notch receptors and ligands to activate lateral inhibition [8,9]. Evidence suggests that Notch signaling plays a pivotal role in fibrosis. Notch1, for instance, is activated in the early stages of fibrosis and induces alveolar epithelial cell proliferation, particularly in type II alveolar epithelial cells (AEC2), contributing to fibrosis development [10]. Notch3, on the other hand, has been shown to mitigate the decline in lung function in IPF mice by promoting fibroblast activation, collagen deposition, and airway basal cell differentiation, all of which exacerbate fibrosis [11]. Conversely, the loss of Notch3 slows the progression of pulmonary fibrosis [12]. However, research into the diagnostic potential of Notch-related genes and their regulatory mechanisms in IPF remains insufficient.

This study aims to investigate Notch signaling pathway genes in relation to IPF, identify potential therapeutic targets, and expand the range of treatment options and strategies for managing this debilitating disease.

## 2. Methods

### 2.1 Data acquisition, differential analysis, and WGCNA

IPF-related datasets (GSE28042 and GSE38958) were retrieved from the Gene Expression Omnibus (GEO) database (https://www.ncbi.nlm.nih.gov/gds). No additional normalization was performed, as the data had been pre-standardized by the submitter. The GSE28042 dataset, based on the GPL6480 platform, underwent quantile normalization to correct for technical variations and batch effects between samples. The GSE38958 dataset, based on the GPL5175 platform, was processed using Robust Multi-array Average (RMA) normalization, which includes background correction, quantile normalization, and probe set summarization for data standardization. The GSE28042 dataset, which includes blood samples from 19 control and 75 patients with IPF, served as the training set, while GSE38958, containing blood samples from 45 control patients and 70 patients with IPF, was used as the external validation set. A total of 428 Notch pathway-related genes (NPRGs) were obtained from previous reports [13].

Differentially expressed genes (DEGs) IPF and control samples were identified in the GSE28042 dataset using the limma package (v 3.54.0), with criteria of |log2FC| > 0.5 and adj.P.Val < 0.05 [14]. Weighted gene coexpression network analysis (WGCNA) was performed to identify genes associated with IPF. Relevant modules linked to IPF traits were selected via WGCNA (v 1.71) in GSE28042, and genes within these modules were designated as key module genes. Outlier samples were excluded following clustering of all the samples. The soft threshold (β) was determined to ensure the network adhered to a scale-free topology (fit index signed R^2^ ≥ 0.85). Gene distance was measured using the dissimilarity of the topological overlap matrix (TOM) (1-TOM). Gene clustering was then performed using average linkage clustering, and dynamic pruning was applied with the cutreeDynamic function to identify modules, with a minimum module gene count set of 100 and a deepSplit value of 2. Initially identified modules were further clustered, and similar modules were merged using a cutHeight threshold of 0.5. The Pearson correlation between modules and grouped traits was calculated, and significance was validated using the Student’s t-test. Candidate genes were derived by overlapping the key module genes, DEGs, and NPRGs. Gene Ontology (GO) and Kyoto Encyclopedia of Genes and Genomes (KEGG) enrichment analysis of the candidate genes was conducted using ClusterProfiler (v 4.7.1.3).

### 2.2 Acquisition of biomarkers

To identify potential diagnostic genes, LASSO and SVM-RFE, analyses were performed on the candidate genes, using the glmnet (v 4.0−2) and caret packages [15]. In the SVM-RFE analysis, the default radial basis function kernel was applied, and 10-fold cross-validation was carried out by randomly splitting the dataset into 10 subsets. Nine subsets were used for training, and one subset was used for testing, with the best-performing model selected for further analysis. The intersection of the diagnostic genes identified by both machine learning methods was considered a potential biomarker. To assess the ability of these biomarkers to distinguish IPF samples from control samples, receiver operating characteristic (ROC) curves were generated using the pROC package (v 1.18.0) for both the training set and the external validation set [16]. A higher area under the curve (AUC) indicated more accurate gene identification. Additionally, biomarker expression was validated in IPF and control samples from the GSE28042 and GSE38958 datasets. The GeneMANIA database (http://genemania.org) was used to explore regulatory relationships between biomarkers and their interacting genes, as well as for enrichment analysis. To investigate pathways associated with the biomarkers, gene set enrichment analysis (GSEA) was performed on the basis of KEGG gene sets using ClusterProfiler (v 4.7.1.3) in the GSE28042 dataset. Ingenuity Pathway Analysis (IPA) was also conducted to identify relevant pathways.

### 2.3 Establishment of nomogram and immune analysis

A nomogram for the biomarkers was constructed in the GSE28042 dataset using the rms package (v 6.5−0)), and calibration curves were plotted to assess the predictive accuracy of the nomogram. Furthermore, the CIBERSORT algorithm (v 2.0.4) was used to calculate the abundance of 22 immune cell types in the training set samples [17]. Differentially expressed immune cells were identified using the Wilcoxon test between IPF and control samples, and correlations between biomarkers and differentially expressed immune cells, as well as among immune cells, were assessed using Spearman correlation. A |cor| > 0.3 and P < 0.05 were considered significant.

### 2.4 Correlation analysis between IPF and activation of oxidative stress, EMT, and TGF-β

Given that IPF is associated with the activation of oxidative stress, EMT, and transforming growth factor-beta (TGF-β) pathways., the pathway scores for each sample in the GSE28042 dataset were calculated using the GSVA package (v 3.18) [18]. The correlation between biomarker expression and pathway scores was then determined. Oxidative stress-related genes were retrieved from the Amigo database (http://www.eecs.qmul.ac.uk/mmv/datasets/amigos/index.html), EMT pathway-related genes were obtained from the dbEMT2 database (http://dbemt.bioinfo-minzhao.org/download.cgi), and TGF-β pathway-related genes were sourced from previously published literature [19].

### 2.5 Construction of regulatory networks and drug prediction

The miRNA prediction for biomarkers was carried out using the miRDB (https://mirdb.org/),miRTarBase (http://mirtarbase.mbc.nctu.edu.tw/index.html), and miRWalk (http://mirwalk.umm.uni-heidelberg.de/) databases. Candidate miRNAs were obtained by overlapping the predicted miRNAs for each gene from the three databases. Transcription factor (TF) prediction for the biomarkers was conducted using the ChEA3 database (https://jaspar.genereg.net/). A TF‒mRNA‒miRNA regulatory network based on the top 50 ranked TFs and candidate miRNAs was constructed using Cytoscape software. Potential small-molecule drugs targeting the biomarkers were predicted via the DGIdb database (https://dgidb.org/), and biomarker–drug networks as well as miRNA–mRNA–TF regulatory networks were visualized in Cytoscape.

### 2.6 RT-qPCR analysis

RT-qPCR validation was performed using blood samples from human subjects to confirm the expression of the biomarkers. Total RNA was extracted from 10 samples (5 control samples and 5 IPF samples) using TRIzol reagent (Ambion, Austin, USA), strictly following the manufacturer’s instructions. The extracted RNA was transcribed into cDNA using the SureScript first-strand cDNA synthesis kit before proceeding to qPCR. qPCR was conducted using 2x Universal Blue SYBR Green qPCR Master Mix. The GAPDH gene was used as an internal control, and the relative expression of biomarkers was determined using the 2^-∆∆Ct^ method. All measurements were performed in triplicate to ensure accuracy and reproducibility. Primer details are provided in **Table 1**.

**Table 1:** RT-qPCR primer list.

Primer	Sequence
GATA2-F	GTCCGAACCATCCCAACCC
GATA2-R	GTAGGAGCTGGGGGTAGAGT
IL4-F	CCACGGACACAAGTGCGATA
IL4-R	CTCTCTGGGCTTTGTAGGCG
NBEA-F	AGGACCAGAAGCAGTTCGTG
NBEA-R	GCGGACTCTGTGGAAGGAAA
PLXND1-F	ACTGTGGGAAACTGATGGGG
PLXND1-R	AGGCGATGCTGGTATCTGTG
GAPDH-F	CGAAGGTGGAGTCAACGGATTT
GAPDH-R	ATGGGTGGAATCATATTGGAAC

### 2.7 Ethics declarations

This study was conducted in accordance with the Declaration of Helsinki, and approved by the Institutional Review Board (or Ethics Committee) of the Affiliated Hospital of Shandong University of Chinese Medicine (protocol code: (2023) Ethical Review No. (065) – KY, approval date: 2023-06-20). Recruitment occurred from July to August 2023, and written informed consent was obtained from all participants. No minors were involved..All procedures adhered to applicable laws and ethical guidelines, with informed consent obtained from participants and/or their legal guardians.

### 2.8 Statistical analysis

Data analysis was performed using R (version 4.1.1, https://www.r-project.org/), and intergroup differences were assessed using the Wilcoxon test, with P < 0.05 considered statistically significant. For qRT-PCR experiments, each sample was tested in triplicate, and three distinct sample groups were used for validation.

## 3. Results

### 3.1 Seven Notch signaling-related genes were identified in IPF

**Fig 1:**
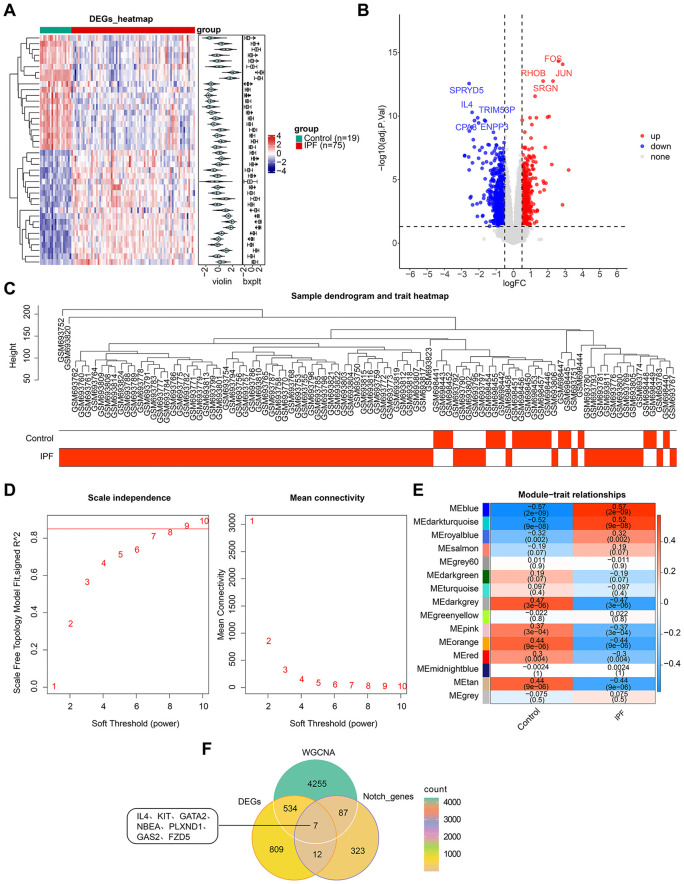
Notch-associated differentially expressed genes between IPF and healthy samples. (A) Heatmap of DEGs between the IPF and control groups. (B) Volcano plot of DEGs between the IPF and control groups. (C) Sample clustering diagram: branches represent samples, with the ordinate indicating the height of hierarchical clustering. (D) Determination of the optimal soft threshold: determination of the soft threshold of for the data. (E) Heatmap showing the relationship between gene modules and traits with sample grouping as phenotypes. (F) Venn diagram displaying the seven candidate genes obtained from the intersection.

A total of 1,361 DEGs were identified in IPF, including 548 upregulated genes and 813 downregulated genes (Fig 1A-1B). Sample clustering revealed the exclusion of two outlier samples from the study (**Fig 1C**). The network demonstrated a scale-free distribution with a power of 9 (**Fig 1D**). A significant positive correlation was observed between the blue module, containing 4,883 genes (cor = 0.57, P < 0.05),and IPF, identifying it as a key module (**Fig 1E**). Seven candidate genes were derived by overlapping the key module genes, DEGs, and NPRGs (**Fig 1F**).

**Fig 2:**
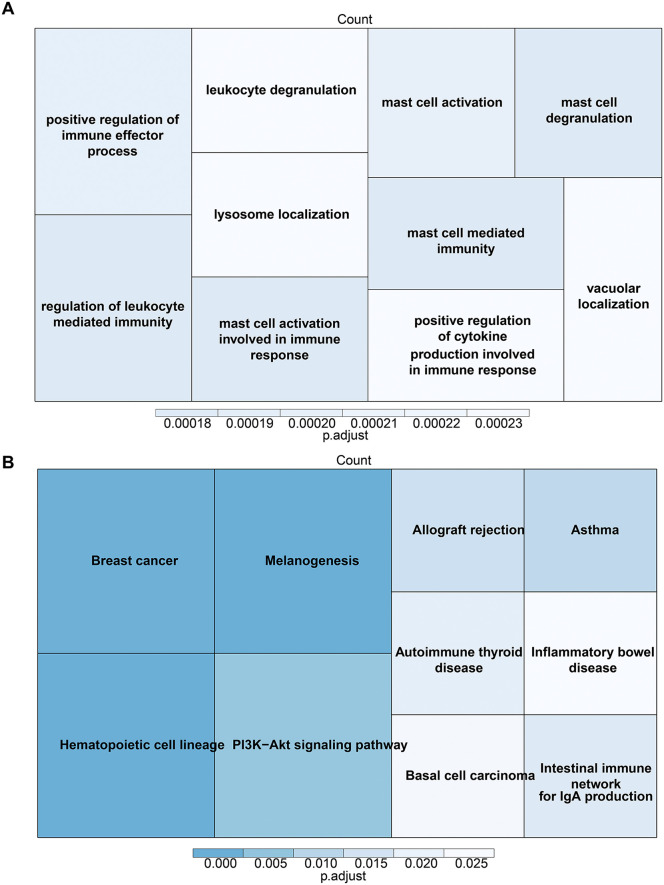
Enrichment analysis of candidate genes. (A) Gene Ontology (GO) enrichment analysis of candidate genes. (B) Kyoto Encyclopedia of Genes and Genomes (KEGG) enrichment analysis of candidate genes. The size of the block represents the number of enriched genes within each pathway, and the color intensity indicates the significance of the pathway enrichment. Larger blocks and darker colors correspond to more significant enrichment.

Additionally, enrichment analysis of the candidate genes revealed their involvement in mast cell activation, immune response, mast cell degranulation, and positive regulation of immune effector processes, as indicated by Gene Ontology (GO) and Kyoto Encyclopedia of Genes and Genomes (KEGG) pathways (Fig 2A-2B).

### 3.2 Machine learning diagnostic model of IPF

**Fig 3:**
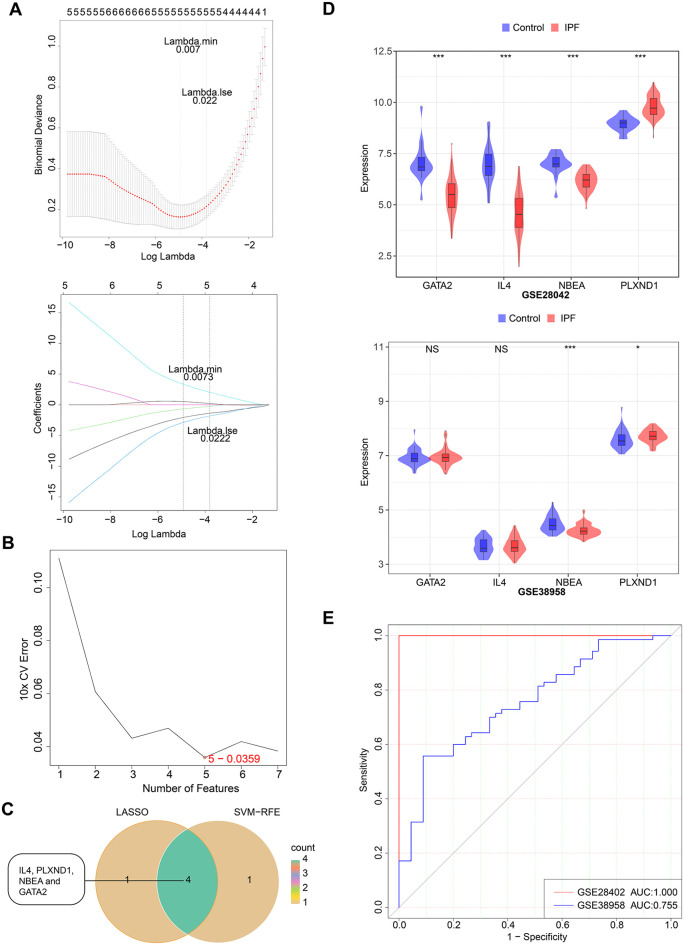
Identification of four biomarkers. (A) Least absolute shrinkage and selection operator (LASSO) regression analysis. The left ordinate represents the local likelihood deviation, and the right ordinate represents the gene coefficients. (B) Support vector machine recursive feature elimination (SVM‐RFE) algorithm to identify the optimal combination of model genes. (C) Venn diagram of the biomarkers. (D) Expression validation chart of the biomarkers in the training set and external validation sets. (E) ROC curve of the diagnostic model in the training set and external validation sets.

To evaluate the diagnostic potential of IPF-associated candidate genes, machine learning models were constructed and validated using LASSO analysis and the SVM-RFE algorithm. LASSO regression analysis identified five model genes (*IL4*, *GATA2*, *NBEA*, *PLXND1*, and *FZD5*) with regression coefficients approaching zero at λ = 0.007 after 10-fold cross-validation (**Fig 3A**). The SVM-RFE model also identified five model genes (*IL4*, *PLXND1*, *NBEA*, *GATA2*, and *GAS2*), with the predicted true value change curves plotted (**Fig 3B**). FZD5 is a key component in mediating the interaction between TGF-β1 and the non-canonical Wnt pathway, influencing IPF-associated epithelial cell dysfunction via sFRP2 as an intermediary [20]. Although FZD5 and GAS2 were identified as model genes, current research does not establish a direct link between them and IPF. Overlap of the model genes identified four key biomarkers (*IL4*, *PLXND1*, *NBEA*, and *GATA2*) (**Fig 3C**). Using these four NPRGs, an IPF diagnostic model was developed. ROC curves for GSE28402 (AUC = 1, sensitivity = 1, specificity = 1) and GSE38958 (AUC = 0.755, sensitivity = 0.557, specificity = 0.911) validated the model’s reliability in predicting IPF (Fig 3D-3E).

### 3.3 PPI network and enrichment analysis

**Fig 4:**
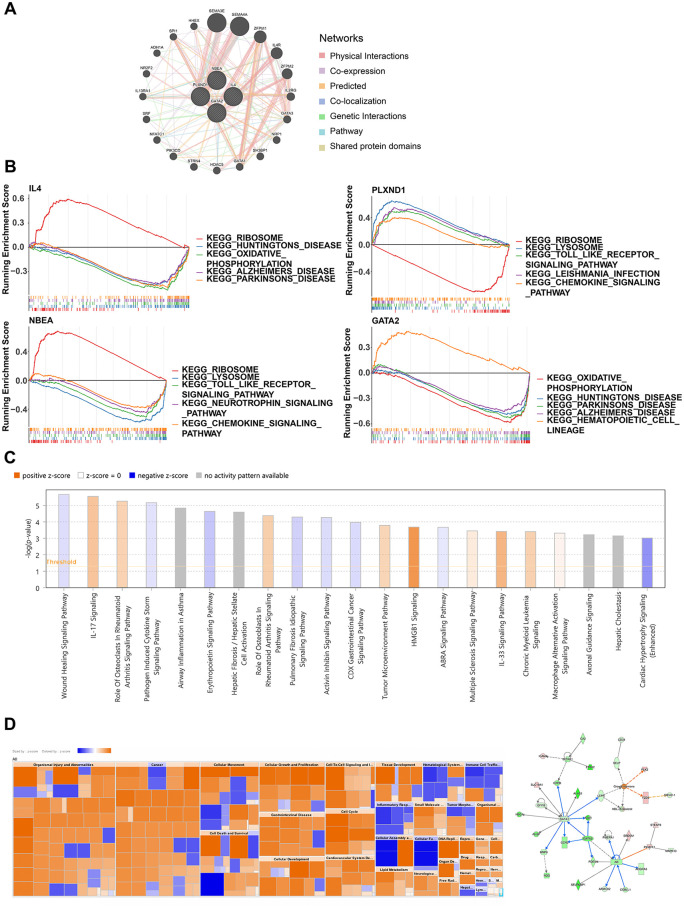
Functional enrichment analysis. (A) Construction of the gene‒gene interaction network and associated pathways. (B) Single-gene gene set enrichment analysis (ssGSEA) of biomarkers. (C) Ingenuity pathway analysis (IPA) of the four biomarkers. (D) Biomarker-disease association heatmap and regulatory networks involving biomarkers.

A gene‒gene interaction network was constructed (**Fig 4A**), revealing that genes such as *SPI1*, *HHEX*, and *SEMA3E* interact with the biomarkers, with most of these interactions being direct physical effects. Functional pathway enrichment analysis highlighted certain commonalities and potential biological associations between the biomarkers. Specifically, IL4 and GATA2 are associated with oxidative phosphorylation and multiple neurodegenerative disease pathways, suggesting a potential synergistic or complementary role in energy metabolism and nerve cell function. Both PLXND1 and NBEA were significantly enriched in the chemokine signaling pathway, and both were implicated in the lysosome and Toll-like receptor signaling pathways (**Fig 4B**). These findings indicate that these genes may share functions in immune response and intracellular signaling. IPA analysis identified 41 pathways in which the biomarkers may be involved, including the IL-17 signaling and WHP signaling pathways (**Fig 4C**). The heatmap revealed that these genes participate in a variety of biological processes, including cell proliferation, immune response, substance metabolism and tissue development, and are significantly activated or inhibited in multiple disease pathways (**Fig 4D**).

### 3.4 Immune cell infiltration results and correlation analysis with pathways

**Fig 5:**
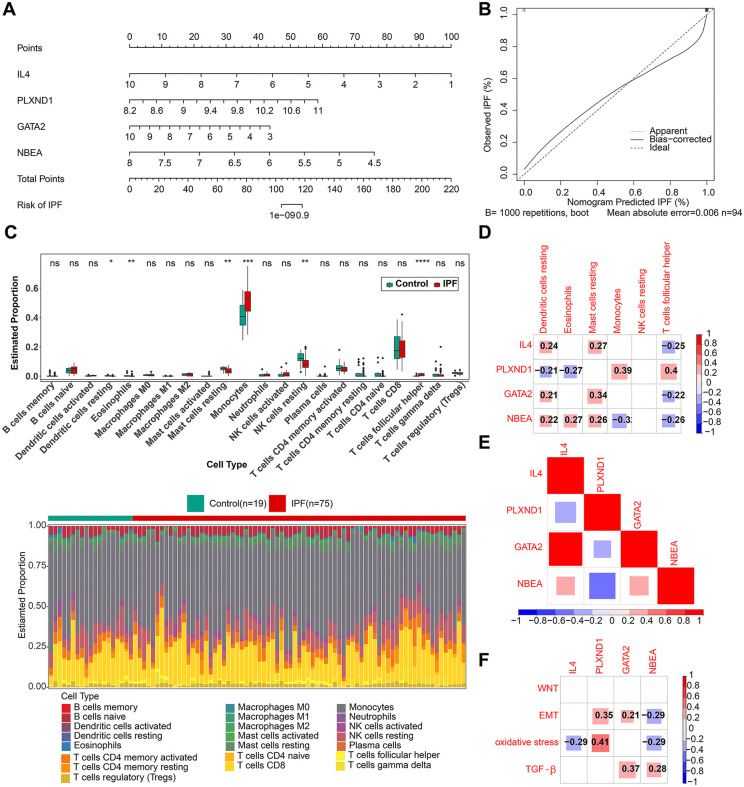
Construction of the nomogram and immune correlation analysis. (A) Nomogram constructed from biomarkers. (B) Calibration curves for nomogram prediction. (C) Box plot of IPF and control group expression across different cell types and heatmaps showing the proportions of immune cells in IPF and control groups. (D) Heatmap showing correlations between biomarkers and differentially expressed immune cells. (E) Heatmap of correlations between biomarkers. (F) Heatmap of correlations between biomarkers and oxidative stress, epithelial–mesenchymal transition (EMT), and transforming growth factor beta (TGF-β).

To better visualize the relationships between the four biomarkers and IPF, a nomogram was developed using multiple logistic regression (**Fig 5A**). The calibration curve indicated that the nomogram demonstrated excellent diagnostic performance (**Fig 5B**). Six differentially expressed immune cell types (eosinophils, resting NK cells, resting mast cells, follicular helper T cells, resting dendritic cells, and monocytes) were identified from IPF and control samples in GSE28042 (**Fig 5C**). *IL4*, *NBEA*, and *GATA2* were strongly positively correlated with resting dendritic cells and negatively correlated with T follicular helper cells. Conversely, *PLXND1* showed a negative correlation with resting dendritic cells and a positive correlation with T follicular helper cells (**Fig 5D**). Correlation analysis revealed a significant negative association between *PLXND1* and the other biomarkers (**Fig 5E**). Additionally, analysis of the biomarkers’ correlation with oxidative stress, EMT, and TGF-β pathways showed the strongest positive correlation between PLXND1 and oxidative stress (**Fig 5F**).

### 3.5 Construction of a TF‒miRNA regulatory network and prediction of small molecule drugs

**Fig 6:**
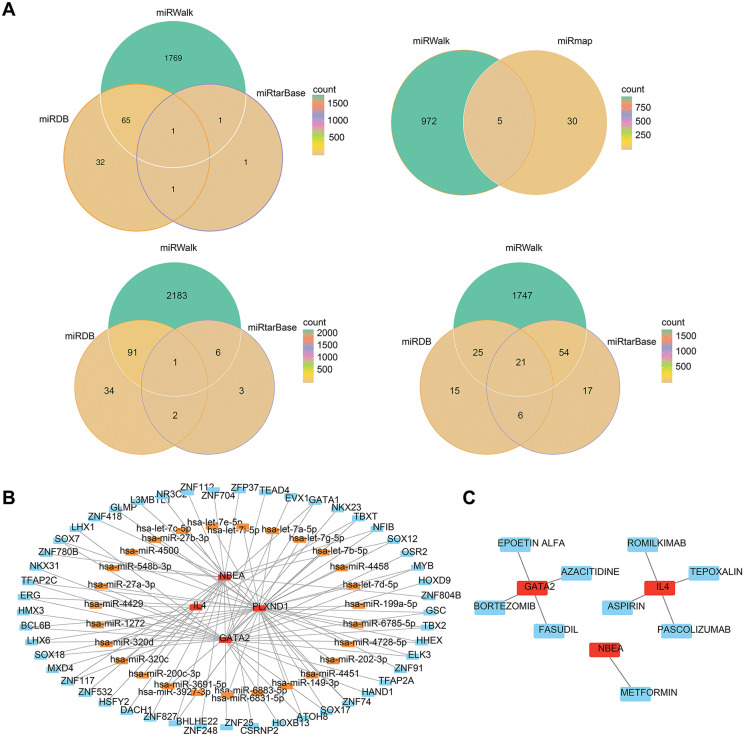
Network construction. (A) Venn diagram showing the intersection of miRNAs predicted for *GATA2*, *IL4*, *NBEA* and *PLXND1* across the three databases. (B) Construction of the TF-mRNA‒miRNA regulatory network. Red represents biomarkers, orange indicates miRNAs, and blue indicates transcription factors. (C) Construction of the biomarker‒drug network. Red indicates biomarkers, and blue represents drugs.

A total of 28 candidate miRNAs were identified by intersecting the results from the three databases (**Fig 6A**). A TF‒mRNA‒miRNA regulatory network was constructed based on the top 50 ranked TFs and candidate miRNAs (**Fig 6B**). The network consisting of 80 nodes and 98 edges suggested that NBEA may regulate hsa-miR-548b-3p through its influence on ZNF780B. Nine potential small-molecule drugs (**S1 Table**), including EPOETIN ALFA and FASUDIL, were identified, as possible modulators of biomarkers, and a biomarker‒drug network with 12 nodes and 9 edges was constructed (**Fig 6C**).

### 3.6 The expression levels of biomarkers

**Fig 7:**
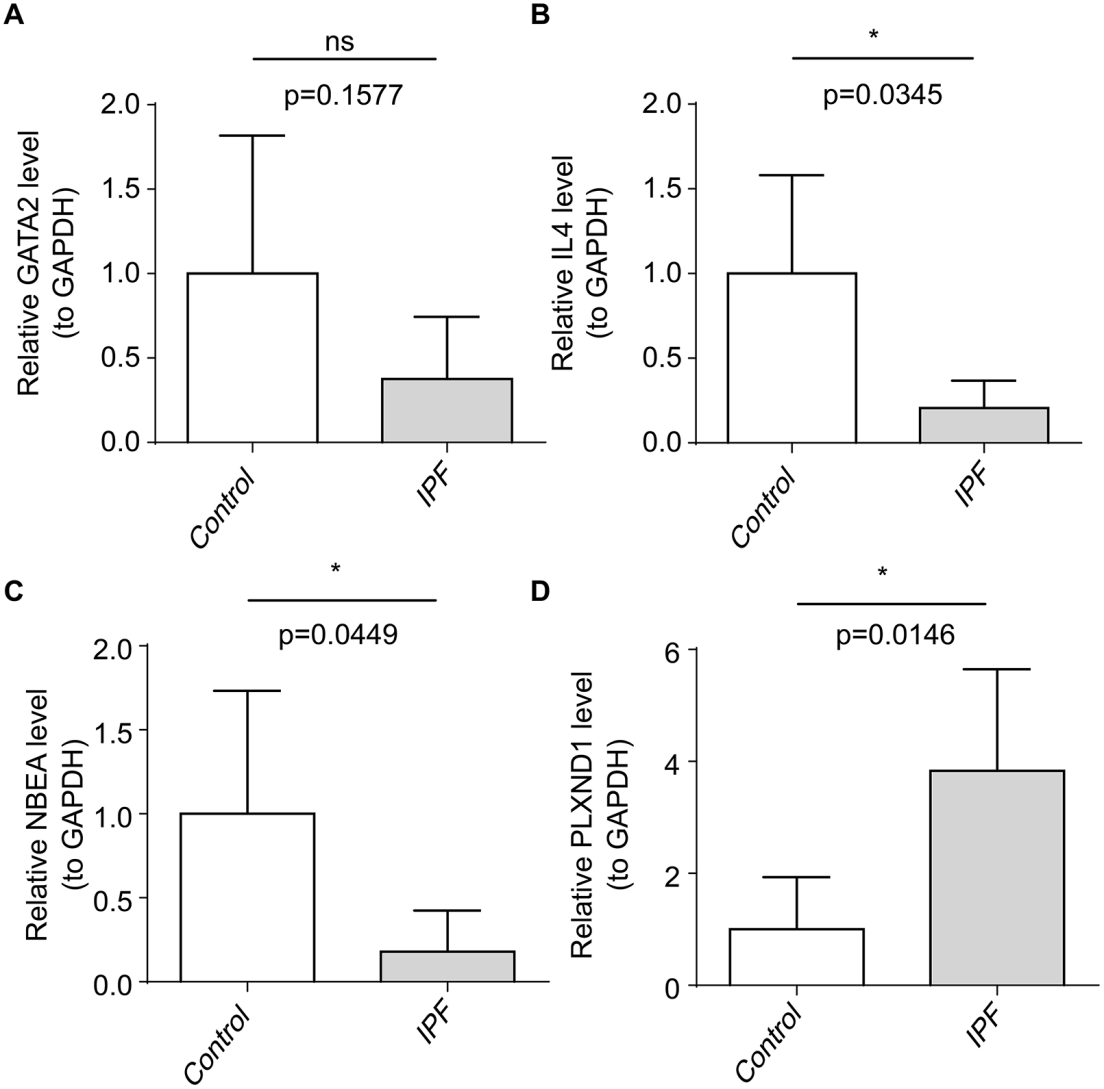
Real-time quantitative polymerase chain reaction (RT‒qPCR) for the validation of biomarker expression. (A) *GATA2* gene; (B) *IL4* gene; (C) *NBEA* gene; (D) *PLXND1* gene. ns, not significant; *, p < 0.05; **, p < 0.01; ***, p < 0.001; ****, p < 0.0001.

Real-time quantitative polymerase chain reaction (RT‒qPCR) analysis confirmed that the trends in expression of *NBEA* and *PLXND1* in the IPF and control groups mirrored those observed in the training and validation sets. Specifically, *NBEA* expression was significantly downregulated, while *PLXND1* was markedly upregulated (Fig 7). *GATA2* and *IL4* also exhibited trends consistent with the training set, although *GATA2* did not show significant differences between groups. This lack of significance may be due to the small sample size or the complex regulatory mechanisms governing *GATA2* expression in different individuals or conditions. Therefore, further validation with larger sample sizes is recommended.

## 4. Discussion

IPF is a progressive, refractory lung disease with a poor prognosis and limited treatment options. The Notch signaling pathway, a highly conserved pathway in mammals, plays a pivotal role in pulmonary fibrosis [21]. However, systematic research on the role of Notch signaling in the regulation and diagnosis of IPF remains limited. To address this gap, this study analyzed IPF transcriptome data from the GEO database. Through differential expression analysis and WGCNA, biomarkers including *IL4*, *PLXND1*, *NBEA* and *GATA2*, were identified and further screened using a machine learning algorithms. Notably, this study primarily reveals correlations rather than causal relationships. While the expression changes of these genes are associated with IPF status, whether they directly contribute to the pro-fibrotic process remains to be confirmed by functional experiments.

Recent studies have highlighted the pivotal role of the NOTCH pathway, particularly NOTCH3 signaling, in the pathogenesis of IPF. NOTCH3 may accelerate IPF progression by regulating the activities of fibroblasts and lymphatic endothelial cells [22]. The NOTCH3 pathway is central to fibroblast activation and transdifferentiation, influencing the repair and fibrotic processes in the lungs [11]. Deletion of NOTCH3 has been shown to significantly reduce the onset and progression of pulmonary fibrosis, suggesting a potential target for IPF treatment [12].

Previous research has demonstrated that IL4 induces M2 macrophage polarization, which is typically associated with pro-fibrotic responses [23]. Therefore, the downregulation of IL4 observed in this study may reflect impaired anti-fibrotic responses or indicate a unique role of IL4 in IPF pathogenesis, warranting further investigation. As key innate immune cells involved in pulmonary fibrosis, macrophage activation is influenced by the Notch signaling pathway. *IL4* has been shown to activate Notch signaling in human macrophages through the Notch ligand Jagged1 [24]. These findings suggest that *IL4* regulates macrophage phenotypic transformation via the Notch signaling pathway and plays a pro-fibrotic role in IPF.

*PLXND1*, a member of the semaphorin receptor family, is dynamically expressed in various embryonic tissues and functions as a serotonin receptor. It plays a critical role in processes such as cell proliferation, migration, and immune regulation [25]. In IPF, the Notch signaling pathway, as a key upstream transcriptional regulator, may directly activate the expression of *PLXND1* [26]. As a functional effector of Notch signaling, *PLXND1*, through binding to its ligand P61-Sema3E, triggers downstream pro-fibrotic signals: it activates ErbB2 phosphorylation to promote fibroblast proliferation and migration, and it may also enhance the fibrotic process by inducing Slug-mediated EMT [27].

GATA binding protein 2 (*GATA2*) is a zinc finger TF primarily involved in proliferation, immune response, and the proliferation of vascular smooth muscle cells [28,29]. The Notch signaling pathway regulates *GATA2* expression via an incoherent feedforward loop: it not only enhances *GATA2* transcription but also initiates a suppressive feedback loop through the induction of HES1 [30]. Dysregulation of this pathway can lead to excessive *GATA2* expression, which activates fibroblasts and increases extracellular matrix accumulation by elevating miR-409-3p levels. This dysregulation impairs the phagocytic function of alveolar macrophages, thereby promoting the progression of pulmonary fibrosis.

*NBEA* has been identified as a novel tumor suppressor gene, and the pathogenesis of IPF shares similarities with cancer, particularly in genetic expression, signaling pathways, and cellular senescence [31]. Mutations in *NBEA* have been observed in patients with cancer-associated fibroblast (CAF) activation [32]. These findings suggest that NBEA may also play a role in the development of pulmonary fibrosis, offering a new perspective for further research into its involvement in the disease.

Biomarkers such as *IL4*, *PLXND1*, *GATA2*, and *NBEA* regulate the progression of IPF through the Notch signaling pathway, which may provide valuable targets for the development of therapeutic strategies for IPF.

Immune infiltration analysis revealed that *PLXND1* showed the strongest positive correlation with monocytes, with a correlation coefficient of 0.39. *IL4*, *GATA2*, and *NBEA* were significantly positively correlated with resting mast cells and resting dendritic cells, while being negatively correlated with follicular helper T cells. Immune cells play a pivotal role in the development of IPF, influencing disease progression and offering critical insights for therapeutic strategy development. Michael et al. reported that elevated monocyte levels are associated with worsening IPF, higher hospitalization rates, and increased mortality risks. The combined diagnosis of *PLXND1* and monocytes could provide a unique and accurate biomarker for patients with IPF [33]. Resting dendritic cells, as sentinels of the immune system are involved in initiating immune responses [34]. Shimbori et al. found that mast cells may activate the TGF-β1 signaling pathway in pulmonary fibrosis, contributing to the progression of the disease [35]. In patients with IPF, the median proportion of follicular helper T cells among total T cells was significantly increased, with many patients showing an increase in activated Tfh cells in the peripheral blood, potentially linked to the pathological immune response [36]. Mast cells, which contain several profibrotic mediators, including tryptase, histamine, leukotrienes, and transforming growth factors, which contribute to creating a profibrotic environment that drives IPF progression. The results of our analysis align with these findings. Therefore, it is hypothesized that *IL4*, *GATA2*, *NBEA*, and *PLXND1* may influence IPF development by regulating these immune cell populations.

miRNAs are a class of evolutionarily conserved small noncoding RNAs that regulate gene expression by degrading messenger RNA (mRNA) or suppressing its protein translation. These molecules play critical roles in various biological processes, including cell differentiation and proliferation. In recent years, the role of miRNAs in the pathogenesis of IPF has gained increasing attention [37]. The TF-mRNA‒miRNA network analysis indicated that miR-200c-3p may target IL4 and contribute to the development of pulmonary fibrosis. miR-200c-3p inhibits EMT by negatively regulating ZEB1, thereby playing a key antifibrotic role in IPF [38]. Additionally, miR-let-7d-5p was found to target PLXND1. Research by Pandit et al. showed that miR-let-7d is significantly reduced in the IPF alveolar epithelium, and downregulation of this miRNA leads to upregulation of other fibrosis-related targets. Inhibition of miR-let-7d promotes the expression of mesenchymal markers in lung epithelial cells, thus impeding pulmonary fibrosis progression [39]. miR-27a-3p inhibits the differentiation of lung myofibroblasts *via* a negative feedback mechanism induced by TGF-β1, blocks SOX18 expression, induces apoptosis, and reduces TGF-β1 expression [40]. TGF-β1 is considered one of the most important profibrotic mediators in IPF development, and miR-27a-3p may also target GATA2 during the process of pulmonary fibrosis(20) [41]. The construction of TF–mRNA–miRNA network enhances understanding of the upstream and downstream regulatory pathways involved in pulmonary fibrosis, potentially leading to novel diagnostic and therapeutic strategies for IPF and other interstitial lung diseases.

Furthermore, several small-molecule drugs with potential sensitivity in patients with IPF have been predicted, including fasudil, metformin, bortezomib, and romiplostim (**S1 Table**). Fasudil reverses pulmonary fibrosis by inhibiting the ROCK signaling pathway and blocking the vicious cycle of myofibroblast contraction and extracellular matrix hardening [42]. Metformin, either alone or in combination with adipose-derived mesenchymal stem cells (ADMSCs), promotes tissue regeneration while reducing inflammation, oxidative stress, and fibrosis, thereby alleviating the fibrotic process [43]. Bortezomib, a proteasome inhibitor, significantly reduces bleomycin-induced pulmonary fibrosis in mice by depleting plasma cells [44]. It also limits TGF-β1 expression, helping prevent fibrosis. However, a phase II clinical trial of romiplostim for IPF in 2017 (NCT02345070) revealed that romiplostim did not significantly improve disease progression in patients [45].

This study has some minor limitations. Firstly, it did not incorporate additional IPF cohorts from the GEO database into the analysis. This study intentionally focused the bioinformatics analysis on the transcriptome data from peripheral blood samples. While there are IPF cohorts based on lung tissue available in the GEO database, focusing on homogeneous blood samples is crucial for constructing models with non-invasive diagnostic potential. This approach minimizes the confounding effects of tissue heterogeneity and complex microenvironments, allowing for the identification of stable and reliable biomarker signals in the circulatory system. Future studies could validate the predictive targets identified here and assess their consistency across different patient groups by integrating datasets from multiple sources. Secondly, the relatively small validation sample size may limit the generalizability of the findings. Expanding the sample size in future research will help further validate the potential of these biomarkers. Additionally, the limited sample size and the lack of significant changes in GATA2 expression may restrict its utility as a biomarker, necessitating further functional validation. Future investigations will treat fibroblasts and macrophages with known GATA2 agonists or antagonists to explore its functional effects. Furthermore, ELISA technology will be used to assess secreted proteins, providing more reliable validation data. Finally, since this study has not fully assessed whether these biomarkers can accurately predict both early and late stages of IPF progression, future research should validate their predictive abilities using large-scale clinical data and longitudinal studies. While the discovery of these biomarkers offers new therapeutic strategies and research directions, caution is warranted, as their translation from laboratory research to clinical application requires extensive validation.

In conclusion, this study identified four potential IPF biomarkers related to the Notch signaling pathway through bioinformatics analysis and explored their mechanisms through correlation analysis. *IL4*, *PLXND1*, *NBEA* and *GATA2* show promise as biomarkers for the future treatment of fibrotic diseases, providing new avenues for exploration. However, it should be emphasized that this is a preliminary result, and further scientific verification and clinical trials are needed to confirm their feasibility and effectiveness. Given the small sample size in the training set and the reliance on correlation analysis, the conclusions require further validation through larger-scale functional studies.

## Supporting information

S1 Table Detailed information on 9 potential small-molecule drugs. (XLSX)
